# Synovial chondromatosis of the distal radioulnar joint—a rare case

**DOI:** 10.1093/jscr/rjae394

**Published:** 2024-06-03

**Authors:** Sinan Mert, Tim Nuernberger, Nina Hesse, Elisabeth M Haas-Lützenberger

**Affiliations:** Division of Hand, Plastic and Aesthetic Surgery, LMU University Hospital, LMU Munich, Munich 80336, Germany; Division of Hand, Plastic and Aesthetic Surgery, LMU University Hospital, LMU Munich, Munich 80336, Germany; Department of Radiology, LMU University Hospital, LMU Munich, Munich 80336, Germany; Division of Hand, Plastic and Aesthetic Surgery, LMU University Hospital, LMU Munich, Munich 80336, Germany

**Keywords:** synovial chondromatosis, wrist, distal radioulnar joint, hand surgery

## Abstract

Unclear wrist tumors are a common clinical presentation in patients with pain and movement restrictions of the wrist. We report a rare case of a benign tumor of the wrist in a 63-year-old female patient who presented with a ganglion-like swelling of the right hand. After examination and preoperative radiological diagnostics, the indication for an open surgery was indicated for resection of either a typical ganglion cyst or a peripheral nerve sheath tumor was made. Interestingly, the suspected diagnosis was not correct. The intraoperative finding and histological analysis revealed typical findings of synovial chondromatosis of the distal radioulnar joint. Although synovial chondromatosis is a relatively rare, and even rarer in the wrist, it is important to consider it as a differential diagnosis when a patient presents with a ‘simple’ wrist ganglion.

## Introduction

Synovial chondromatosis (SC) is a rare neoplastic disease of the synovial membrane characterized by cartilaginous and osseous metaplasia. It rarely undergoes malignant transformation. SC almost exclusively affects the knee or hip and less frequently other joints such as the shoulder, elbow, or wrist, with men being more commonly affected than women. Milgram categorized the disease in three phases [1]. The first phase, the ‘active phase’, occurs exclusively in the active synovia without loose bodies. This is followed by the ‘transition phase’, which consists of active intrasynovial proliferation lesions and transitional loose bodies. The third and ‘major phase’ is characterized by the presence of multiple free osteochondral bodies. In this stage, only minimal intrasynovial disease is verifiable. Patients typically suffer from pain, swelling of the joint, and restriction of movement [2]. In case of clinical suspicion, appropriate radiological examination consisting of X-ray and/or MRI must be performed. Treatment of choice is surgical exploration and removal of loose bodies and synovectomy.

We report a rare case of primary SC of the distal radioulnar joint (DRUJ) with an initially uncharacteristic clinical presentation.

## Case Report

A right-handed 63-year-old healthy female secretary presented to our outpatient clinic with a slowly but progressively growing mass on the volar ulnar aspect of her right wrist, along with carpal tunnel syndrome in both hands. She complained about pain that worsened at flexion, permanent swelling, and reduced sensitivity and thus about a certain impairment in everyday life. No similar medical history in any other joint was reported. There wasn’t any history of preceding or repetitive trauma, nor a participation in any kind of sports. The past medical history was unremarkable.

During the physical examination, the patient exhibited swelling and tenderness palmar to the right DRUJ and flexor carpi ulnaris (FCU) tendon. The pain was rated 3 out of 10 at rest and 6–7 out of 10 at stress (numeric rating scale). The preoperatively measured active range of motion (ROM) of the affected wrist was 50° extension, 50° flexion, 5° ulnar deviation, and 20° radial deviation. This represents a restriction in ROM compared to the healthy wrist (70° extension, 80° flexion, 20° ulnar deviation, 30° radial deviation).

Due to a secondary diagnosis of bilateral carpal tunnel syndrome, the patient displayed typical neuropathic sensory abnormalities and neurographical results consistent with median nerve compression.

**Figure 1:**
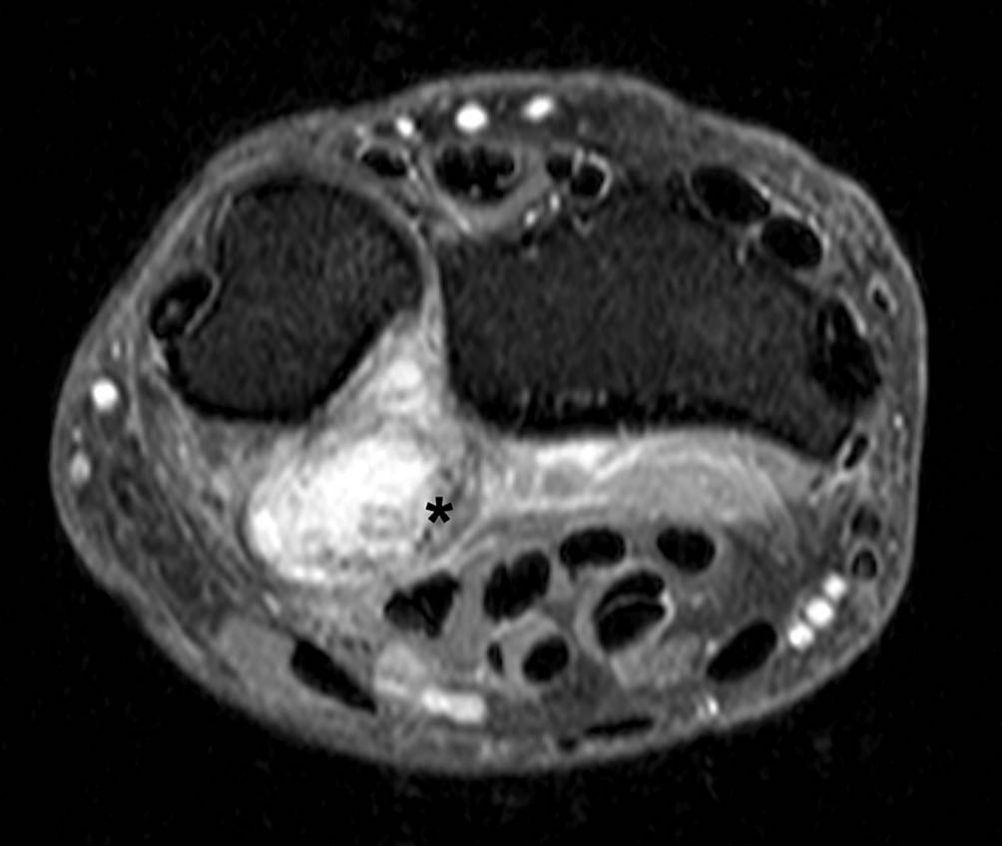
Axial slice of a fat suppressed PD Dixon—synovial capsular proliferation on the volar aspect of the DRUJ (asterix); the capsule is thickened, additionally evidence of hypointense internal structures aside the capsular proliferation.

**Figure 2:**
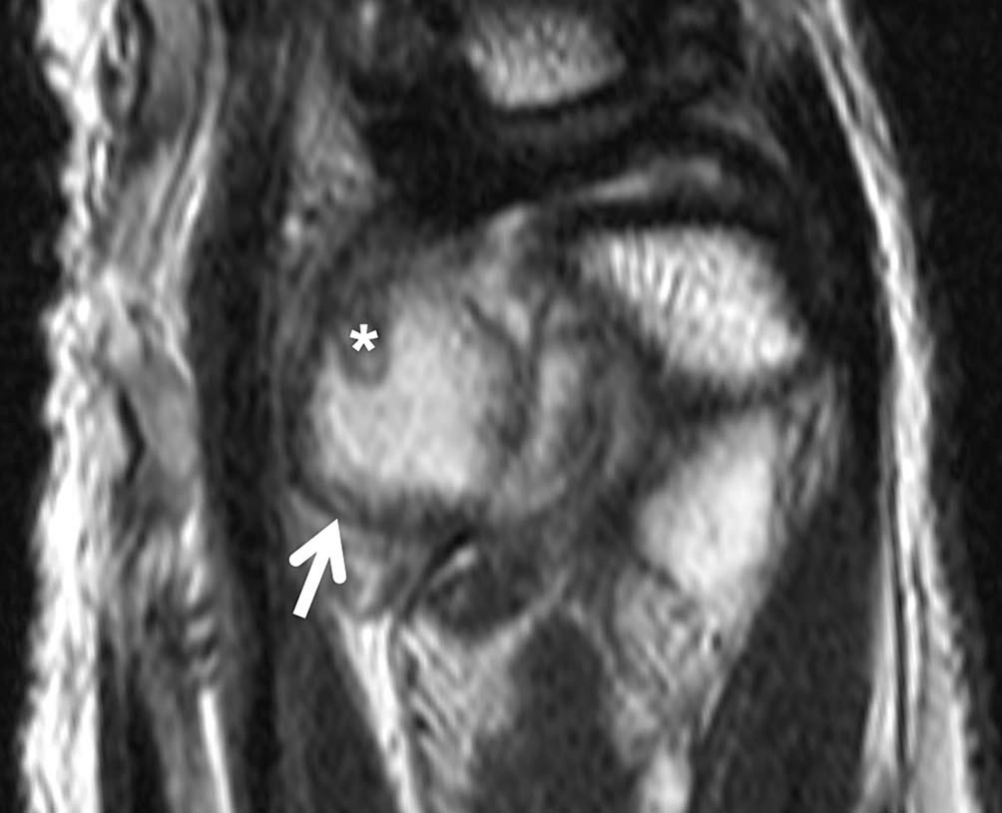
Sagittal slice of a T2-weighted fast-spin echo sequence of the DRUJ—well-defined hyperintense lesion on the volar aspect of the DRUJ; capsular blooming artifact (short arrow); hypointense, internal structure within the distended DRUJ (asterix), chondromatosis foci accordingly.

MRI scan revealed suspicion of ganglion cyst formation. The images (Figs 1 and 2) depicted a palmar-directed mass originating from the DRUJ with a transverse diameter measuring a maximum of 12 x 17 mm. The mass exhibited a predominant fluid isointense internal signal, accompanied by marginal signal depressions on T2-weighted sequence. These depressions within the suspected ganglion raised suspicion of diffuse-type tenosynovial giant cell tumor. The mass had contact to the flexor tendons at the level of the DRUJ. No pathological bone marrow edema was observed.

**Figure 3:**
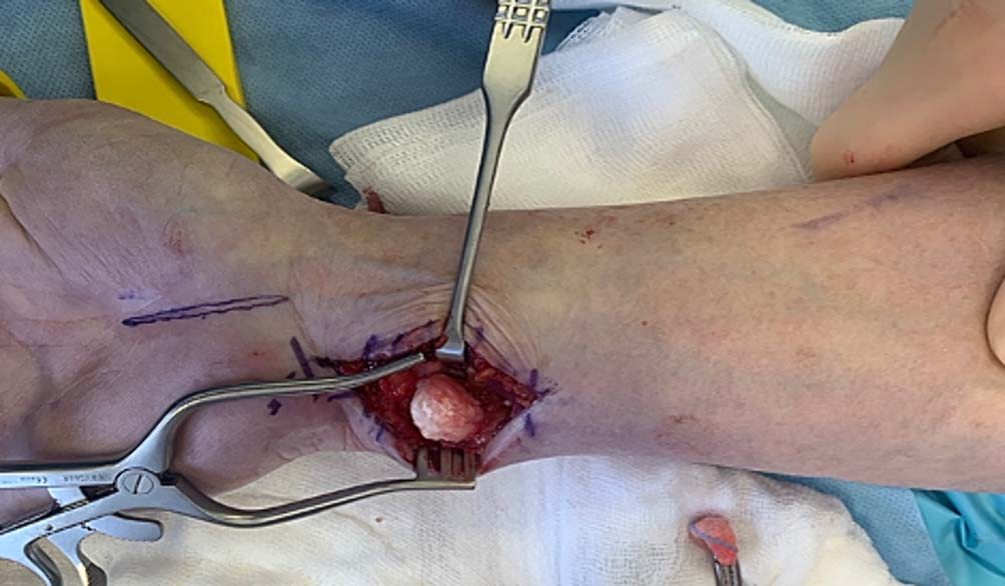
Intraoperative photograph of the exposed SC of the DRUJ—the tumor of 15 mm diameter is located volar to the DRUJ and next to the FCU tendon.

In consideration of the patient’s symptoms and radiological findings, surgical extirpation was performed under axillary plexus anesthesia with an upper arm tourniquet. We performed a longitudinal volar approach, positioned centrally over the palpable tumor mass. Initially, the FCU tendon and the ulnar neurovascular bundle were dissected and protected, allowing exploration of the tumor. A solid mass, lacking typical ganglion characteristics, was dissected, measuring ~15 mm in diameter. The potential origin from the DRUJ was confirmed as the pedicle was followed dorsally. The mass was excised *in toto* without breaching its surrounding capsule (Fig. 3). However, a minimal incision of the DRUJ capsule was necessary to completely dissect the tumor from its origin.

**Figure 4:**
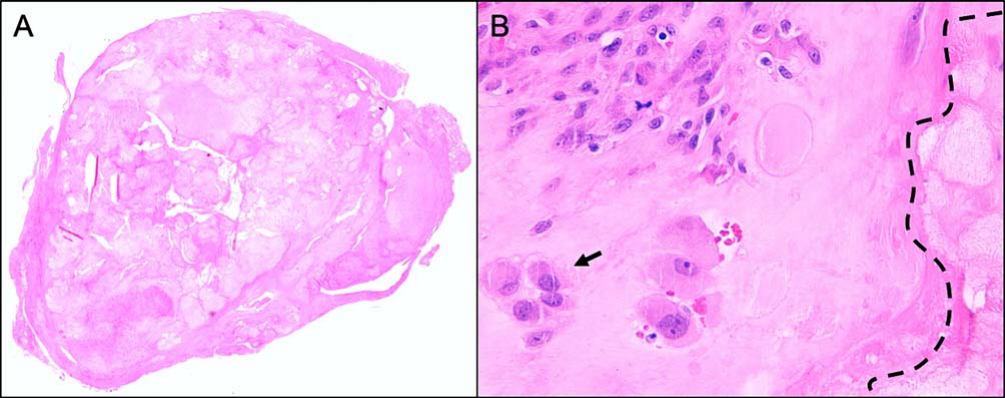
Representative histological slide of the lesion in low magnification (A; 2.5×) shows a relatively well-circumscribed multinodular tumor; higher magnification (B; 40×) demonstrates a cartilaginous nodule, composed of focally clustered chondrocytes (arrow) in a hyaline matrix with beginning calcification (dotted line); both hematoxylin & eosin staining.

The histopathological examination displayed moderately cellular chondroid neoplasia with mild atypia. Along with the localization of the sample, the findings suggested a SC (Fig. 4). Macroscopically, two grayish-beige tissue samples were seen (9 x 15 x 13 mm and 9 x 7 x 3 mm). Several histopathological stains were routinely obtained. Sections showed nodular, centrally partly cystic distended chondroid tissue with predominantly tight fibrous-capsular borders. The tissue was partly nodular chondroid with mature chondrocytes consisting of flattened nuclei and single, well visible nuclei, and on the other hand with spindle-shaped cell proliferates with elongated, partly wave-like pale basophilic nuclei. On examination of 10 high-power fields (HPFs), there was one mitotic figure (1/10 HPF). The S100 expression was positive in both the spindle-cellular component and the chondroid cells, while the p53 expression showed a homogenously distributed and regulated pattern. The Ki-67 immunohistochemistry was 3%.

The postoperative protocol prescribed immobilization in a palmar wrist splint for 2 weeks, followed by the initiation of active and passive ROM exercises under physiotherapeutic guidance.

In the follow-up examination 4 weeks after the surgery, the patient had already regained full ROM. The final examination 1 year after the surgery showed a pain-free patient with no postoperative or recurrent tumor mass swelling. The absence of recurrence was confirmed through sonographic examination.

## Discussion

Our case presented an intra-articular SC with direct infiltration of the DRUJ, where preoperative MRI scans (Figs 1 and 2) did not reveal evidence of calcified bodies, bone bruise, or periosteal reaction. Therefore, the accurate diagnosis was suspected intraoperatively and confirmed by histopathological examination. Similar discoveries during surgery were documented by Misawa *et al*. and Rogachefsky *et al*. in the literature [3, 4]. Kim *et al*. reported in 2015 that no free chondral bodies were found in the surrounding tissue of their case [5].

Our reported case emphasizes that a swollen, tender mass at the volar distal forearm can have various differential diagnoses. These include a typical ganglion cyst, the most common occurrence and the primary working hypothesis in the presented case, a giant cell tumor of the tenosynovium, calcifying aponeurotic fibroma, crystal arthropathy, foreign body, inflammatory arthritis, psoriatic arthropathy, pigmented villonodular synovitis, and degenerative joint diseases.

Primary SC, a benign neoplasia, rarely affects the wrist, hand, or fingers. According to the current literature, the highest incidence of primary SC is found in the fourth and fifth decades with a female-to-male ratio of 1:2 [2]. The most common clinical symptoms are a painless tumor, but tenderness to pressure and compression. Patients with intra-articular chondromatosis specify pain worsening with wrist motion, causing greater psychological strain. Due to the slow growth of the tumor, the first consultation with a physician is around 2 years after the onset of the first symptoms.

In most cases, correct diagnoses were made by preoperative radiological imaging, mostly through MRI scans. However, in some rare cases, such as the presented case or as documented by Rogachefsky *et al*., SC is first diagnosed at surgery [4]. A possible reason could be that typical calcifications, which are evenly distributed and often similar, are only detectable in 70%–95% of primary intraarticular SC [6]. This percentage depends on the current phase of the disease, ranging from 54% in the active phase to 100% in the mature phase [7]. Therefore, in early stages, radiographs may only describe soft tissue swelling. In recent years, CT and MRI scans are used for diagnosing and staging the disease, although in certain cases, as presented in our case with the suspicion of a ganglion, the images can still lead to an incorrect diagnosis. Kramer *et al*. described three different MRI patterns, which are similar in signal intensity to muscle on T1-weighted images [8]. Murphey *et al*. reported the signal intensity of SC to be lower, more similar to fluid on T1-weighted images. When focusing on calcifications, a CT scan is more sensitive for the analysis of bone quality and detection of these pathognomonic findings [6].

The most effective treatment for SC is an open or arthroscopic synovectomy and, if present, excision of the cartilaginous loose bodies. Although Milgram recommend adapting the surgical approach to the phase of the disease, if a patient presents with distinctive symptoms, such as pain and restriction in everyday life, a synovectomy should be performed in any stage [1]. Preoperatively, determining the exact stage of SC is difficult; thus, an operation does not worsen the condition, supported by the fact of the low malignancy rate. The same procedure, open or arthroscopic approach, can be applied for intraarticular chondromatosis, depending on the surgeon’s experience, while surgery on extraarticular chondromatosis should always be performed by an open approach.

Our case highlights the significance of such operations performed by a hand surgeon, experienced enough to correctly treat an intraoperative finding that does not correlate with the initial diagnosis. In our case, total excision without synovectomy was performed in Stage 3 according to Milgram [1]. However, additional synovial cartilage lesions may persist *in situ* and possibly appear as a recurrence postoperatively. Due to the small number of published cases, variety of localization and treatment recommendations, the results are heterogeneous regarding recurrence rates. The recurrence range of intraarticular chondromatosis varies between no differences and major differences between both modalities after an average follow-up of 38 months [7, 9]. Until now, no recurrence has been detected in our patient 1 year postoperatively. This correlates with the literature, which shows that in short-term follow-up periods (mean 32 months, range 6–72 months), a recurrence was registered in about 17% [10].
